# Bismuth Reduces Cisplatin-Induced Nephrotoxicity *Via* Enhancing Glutathione Conjugation and Vesicular Transport

**DOI:** 10.3389/fphar.2022.887876

**Published:** 2022-06-16

**Authors:** Hui Jiang, Yifan Hong, Guorong Fan

**Affiliations:** ^1^ Tongji University School of Medicine, Shanghai, China; ^2^ Institute of Molecular Physiology, Shenzhen Bay Laboratory, Shenzhen, China; ^3^ Department of Clinical Pharmacy, Shanghai General Hospital, School of Medicine, Shanghai Jiao Tong University, Shanghai, China

**Keywords:** cisplatin, bismuth, glutathione, vesicle transport, apoptosis, nephrotoxicity

## Abstract

Bismuth drugs have long been used against gastrointestinal diseases, especially the gastric infection of *Helicobacter pylori*. Cisplatin is a widely used anticancer drug that tends to accumulate at renal proximal tubules and causes severe nephrotoxicity. It was found that bismuth pretreatment reduces cisplatin-induced nephrotoxicity, but the mechanism of action remains unclear. To understand bismuth’s effect on renal tubules, we profiled the proteomic changes in human proximal tubular cells (HK-2) upon bismuth treatment. We found that bismuth induced massive glutathione biosynthesis, glutathione S-transferase activity, and vesicular transportation, which compartmentalizes bismuth to the vesicles and forms bismuth–sulfur nanoparticles. The timing of glutathione induction concurs that of bismuth-induced cisplatin toxicity mitigation in HK-2, and bismuth enhanced cisplatin sequestration to vesicles and incorporation into bismuth–sulfur nanoparticles. Finally, we found that bismuth mitigates the toxicity of general soft metal compounds but not hard metal compounds or oxidants. It suggests that instead of through oxidative stress reduction, bismuth reduces cisplatin-induced toxicity by direct sequestration.

## Introduction

Bismuth compounds are widely used against gastrointestinal illnesses, such as peptic ulcers and *Helicobacter pylori* infection (Li and Sun, 2012). Colloidal bismuth subcitrate (CBS) is a key member of the quadruple therapy against gastric *Helicobacter pylori* infection, which increases the eradication rate of bacteria (Suerbaum and Michetti, 2002; Gisbert, 2011). Bismuth subsalicylate (BSS), the key component of OTC drug *Pepto-Bismol*, cures nausea, diarrhea, and mild stomach upset (Dupont, 1987; Bierer, 1990). Cis-diamminedichloroplatinum (II) (CDDP, or cisplatin) is one of the most used anticancer drugs (Kodama et al., 2014). Clinical use of cisplatin is limited by its dose-limiting toxicity, nephrotoxicity (Lebwohl and Canetta, 1998; Shiraishi et al., 2000). Cisplatin-induced acute kidney injury (AKI) contributes to a large number of hospital-acquired AKI cases, which could then develop into chronic kidney damage (Schetz et al., 2005).

As two most used metallodrugs, an interesting drug–drug interaction exists between bismuth and cisplatin. Bismuth pretreatment minimizes the nephrotoxicity of high-dose cisplatin, without affecting its antitumor effects (Morikawa et al., 1990). Specifically, it prevents cisplatin-induced lethal nephrotoxicity, blood urea nitrogen (BUN) increase, and diarrhea (Hamada et al., 1989). Both bismuth and cisplatin were found in the renal cortex, especially in renal proximal tubules, which provides a solid pharmacokinetic foundation of such a drug–drug interaction (Walker et al., 1990; Leussink et al., 2002). Furthermore, according to the hard–soft acid base (HSAB) theory, both bismuth and platinum are borderline soft metals that have a high binding affinity to thiols. Metallothionein (MT) and glutathione (GSH) are the most important biological thiols that detoxify heavy metals in humans.

MT is a family of low molecular weight cysteine-rich proteins with extremely strong metal binding affinity (e.g., physiologically Zn^2+^ and Cu^2+^ and xenobiotically Bi^3+^, Pt^2+^, and Cd^2+^); it functions as both physiological metal storage protein and heavy metal scavenger (Kagi and Vallee, 1961; Irons and Smith, 1976; Richards and Cousins, 1976; Suzuki et al., 1977; Sugawara, 1978; Leone et al., 1985; Mehra and Bremner, 1985; Sun et al., 1999; Ngu et al., 2010; Miyayama et al., 2011). Bismuth’s induction of MT in renal tissue was proposed as the mechanism behind bismuth’s mitigative effect (Naganuma et al., 1987; Hamada et al., 1989; Naganuma and Imura, 1994; Kondo et al., 2004). However, even though the affinity of platinum (Pt) to MT is approximately 10^7^ times stronger than that of zinc (Zhang et al., 1997), Pt-bound MT in the kidneys of bismuth-pretreated rats is lower than that in the untreated group (Boogaard et al., 1991).

GSH is a simple tripeptide that contains a cysteine residue. GSH is one of the most abundant thiols in mammalian cells, which can conjugate metal ions and neutralize reactive oxygen species (Pljesa-Ercegovac et al., 2008; Jos et al., 2009). Previously, we identified a GSH-mediated intracellular metabolic model of bismuth in cultured proximal tubular cells (Hong et al., 2015). It shows that a significant amount of bismuth was conjugated to glutathione and transported into vesicles by MRP transporter, forming black bismuth particles. Bismuth induces GSH biosynthesis and forms a self-sustained positive feedback circle named sequestration-aided passive transport (SAPT), in which GSH robustly removes bismuth and protects cells from acute toxicity. GSH conjugation and transportation were also reported in cisplatin-resistant cells (Godwin et al., 1992; de Vries et al., 1995), and the consumption of GSH leads to a significant increase in cisplatin toxicity *in vitro* and *in vivo* (Fu et al., 2013). GSH might also serve as an antidote to cisplatin-induced oxidative stress. Whether bismuth-induced SAPT is the major mechanism behind bismuth’s mitigative effect against cisplatin toxicity still remains unclear.

Accumulating evidences from animal studies showed that GSH depletion increased cisplatin nephrotoxicity but reduced cisplatin accumulation in the kidney, and in GSH-depleted rodents, induction of MT mitigates cisplatin nephrotoxicity (Ishikawa et al., 1990; Hanada et al., 2000; Satoh et al., 2000). However, whether it is GSH or MT’s antioxidant or cisplatin-sequestering nature that reduced cisplatin nephrotoxicity remains unknown.

In this report, we thoroughly profiled bismuth’s impacts on the proteome of proximal tubular cells. We found that bismuth increased the levels of cystine importer Slc7a11 and glutathione, the activity of glutathione S-transferase and glutathione reductase, but not the levels of metallothionein isoforms. Using TEM-EDS, we determined that the SAPT-generated bismuth particles are bismuth–sulfur nanoparticles. Bismuth also increased the levels of actin filament and vesicle tethering complex, suggesting enhanced vesicle exocytosis, while in GSH-depleted cells, bismuth greatly induces metallothionein expression, ubiquitin-mediated proteolysis, and DNA mismatch. We successfully reproduced the mitigative effect of bismuth against cisplatin-induced apoptosis in the human proximal tubular cell line; the timing of bismuth pretreatment is in agreement with the activation of GSH metabolism. Also, most of cisplatin absorbed by the cells was incorporated into vesicular bismuth–sulfur nanoparticles, and by systematic comparison, we proved that it is this sequestration mechanism (not antioxidation) by GSH that reduced cisplatin toxicity.

## Materials and Methods

### Cell Culture

HK-2 (ATCC® CRL-2190TM) cells are maintained in Dulbecco’s modified Eagle’s medium (DMEM) containing 10% fetal calf serum (FBS), 100 units/ml penicillin, and 100 μg/ml streptomycin at 37°C in a humidified incubator with 5% CO_2_. The cell culture medium was refreshed every other day. All culture reagents were purchased from Invitrogen Technologies (Carlsbad, CA, United States).

### Inductively Coupled Plasma Mass Spectrometry

Agilent 7500 ICP-MS was used to study the cellular metal content. All cells were washed with PBS, digested with trypsin, and collected by centrifugation at 1,000 rpm for 5 min. The cells were dissolved in 0.2 ml of 50% HNO_3_ and 50% H_2_O_2_ (volume ratio 1:1), incubated at 60°C for 1 h, and diluted to an appropriate concentration. Metal quantification was repeated three times.

### Morphology and Element Identification of Nanoparticles

HK-2 cells were incubated with 500 µM bismuth citrate in a cystine-saturated medium for 48 h. The cells were harvested, lysed in 1% SDS, and centrifuged at 15,000 rpm for 15 min. The pellet was extracted with a two-phase system of 1:2 octanol and water. After 24 h, the particles in the bottom phase of the tube were collected and re-extracted with chloroform. The upper phase was carefully transferred to a new tube and centrifuged (×10,000 g, 30 min). The particles were sequentially washed with ethanol and distilled water and sonicated for 1 h. The morphology and composition of the particles were, respectively, determined by transmission electron microscopy (TEM) and energy-dispersive X-ray spectroscopy (EDS).

### Protein Lysis and Digestion for Proteomics Analysis

The medium was aspirated, and the cells were washed with ice-cold PBS three times. The cells were scraped and collected in 1.5-ml vials. The cells were pelleted by centrifugation for 5 min at ×800 g at 4°C. The cell pellets were snap-frozen in liquid nitrogen and stored overnight at −80°C until further analysis. The pellets were thawed, and the cells were lysed with buffer (Beyotime) 50 mM Tris (pH 7.4), 150 mM NaCl, 1% Triton X-100, 1% sodium deoxycholate, and 0.1% SDS, and supplemented with a complete protease inhibitor mixture (Beyotime). The cells were plated on the ice for 30 min, and sonicated for 15 cycles of 30 s. The total protein concentration was determined using a BCA assay (Thermo). Then, the cells were heated for 5 min at 95°C. Proteins were desalted with acetone and reduced and alkylated with 7M DTT and 1M IAA, respectively. The proteins were digested overnight at 37°C with trypsin (Promega) and with an enzyme-to-substrate ratio of 1:50. The samples were desalted using Sep-Pak C18 cartridges (Waters), eluted with 60% acetonitrile (ACN)/0.1% formic acid (FA), and directly subjected to peptide enrichment. The total peptide concentration was determined using the Pierce™ Quantitative Colorimetric Peptide Assay (Thermo). The samples for the proteome analysis were instead dried down and stored at −80°C until subjected to nLC-MS analysis.

### Reverse Phase Chromatography and Mass Spectrometry

Peptides were subjected to reverse phase nLC-MS/MS analysis using an Easy nLC1200 system coupled to a Q Exactive HF-X mass spectrometer (Thermo Fisher Scientific) for proteome analysis. The chromatograph system was equipped with a trap column (Acclaim PepMapTM 100, 75 μm × 2 cm, C18, 3 μm, 100 Å) and an analytical column (Acclaim PepMapTM RSLC, 75 μm × 25 cm, C18, 2 μm, 100 Å). Trapping was performed for 3 min in solvent A (0.1% FA in water) at 2 μl min−1, while for the elution, the flow rate was passively split to 300 nl min−1. The gradient was as follows: 0–1 min, 8% buffer B (0.1% FA in 80% ACN); 1–98 min, 8–28% buffer B; 98–112 min, 28–36% buffer B; 112–116 min, 36%–100% buffer B; and 116–120 min, 100% buffer B. The mass spectrometer was operated via Xcalibur (version 4.1, Thermo Scientific) in the data-dependent mode. The Orbitrap full-scan MS spectra from m/z 350–1800 were acquired at a resolution of 60,000 after accumulation to a target value of 3e6. The maximum ion injection time was 20 ms. Up to 20 most intense precursor ions were selected for fragmentation, with the isolation window set to 1.6 m/z. HCD fragmentation was performed at a normalized collision energy of 28% after accumulation to a target value of 2e5, with the maximum injection time of 25 ms. MS/MS was acquired at a resolution of 15,000. Dynamic exclusion was set to 60 s.

### Mass Spectrometry Data Processing

Proteome Discoverer 2.1 software (Thermo Fisher Scientific) was applied for identification of peptide and protein. The latest Human UniProtKB/SwissProt Release (2018_10 has 551,681 entries) was used for searching MS/MS spectra. Peak lists were generated with a precursor signal-to-noise ratio of 1.5, and the Sequest algorithm was set as default. Trypsin was set as the specific enzyme, and one missed cleavage was tolerated. The precursor ion mass tolerance was set to 7 ppm, and the product ion mass tolerance was set to 0.06 Da. The peptide false discovery rate (FDR) was determined by performing a decoy database search with the target decoy PSM (Peptide Map Matching) validator module. The peptide FDR threshold is 1%.

### Cell Counting Kit-8 Assay

The survival rate of the cell was measured using Cell Counting Kit-8 (Dojindo). HK-2 cells were cultured in a 96-well plate. CCK-8 solution was added to the cell culture medium at a 1: 10 ratio and incubated for 2 h at 37°C. The absorbance at wavelength 450 nm was measured using an automatic detector (Biotek Synergy 2).

### Glutathione Metabolism–Related Assay Kits

5,5-dithiobis (2-nitrobenzoic acid) (DTNB)–based Glutathione Assay Kit (Beyotime, S0052) and Glutathione Reductase Assay Kit (Beyotime, S0055) were used. The Glutathione S-Transferase (GST) Assay Kit was purchased from Sigma (CS0410), and details of all assays can be found in the manufacturer’s protocol.

### Statistical Analysis

The data are expressed as mean ± standard error of mean. Each experiment was repeated at least three times. The statistical significance was determined using Student’s t-test. Statistical significance is *p* < 0.05.

## Results

### Colloidal Bismuth Subcitrate–Induced Proteomic Alterations in Human Proximal Tubular Cells

**FIGURE 1:**
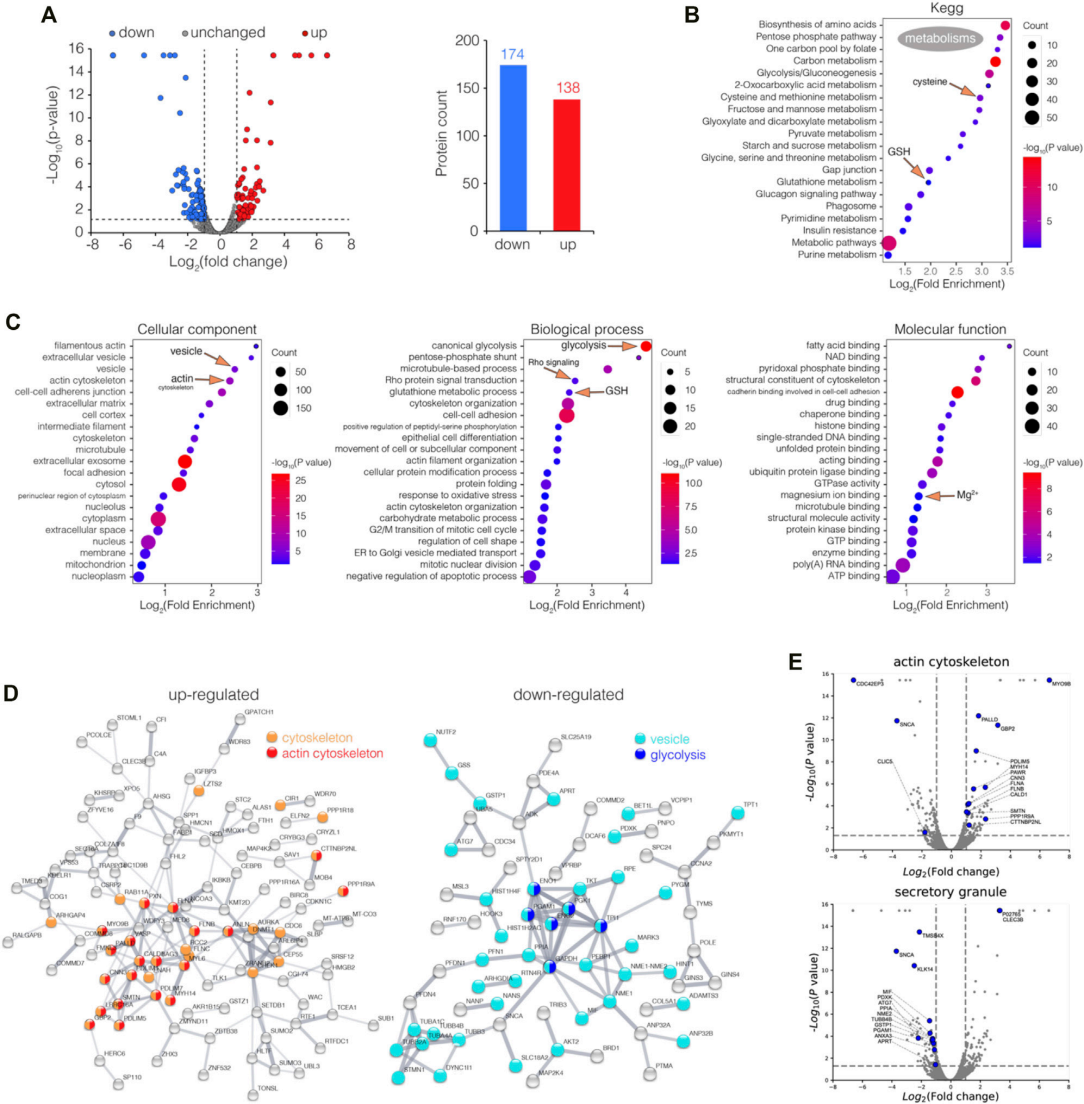
Enrichment analysis of differentially expressed proteins (DEPs) in CBS-treated HK-2 cells. **(A)** Volcano plot visualization of DEPs (fold of change >2 or <0.5, *p* value <0.05) in HK-2 cells treated with 500 μM CBS for 48 h, and 138 and 174 proteins were up and downregulated among 6,230 proteins, respectively. **(B)** Enrichment analysis of DEPs in CBS-treated HK-2 cells, in perspective of KEGG, and **(C)** GO cellular component, molecular function, and biological process. **(D)** Protein–protein interaction networks of both up and downregulated DEPs generated using STRING. **(E)** Volcano plots visualizing DEPs involved in GO terms: actin cytoskeleton and secretory granule. CBS-induced proteomic alterations in GSH-depleted HK-2 cells.

To investigate bismuth’s effect on proximal tubular cells, we studied the proteomic alterations it induces in the proximal tubular cell line HK-2. HK-2 cells were treated with or without 500 μM CBS for 48 h, and the cells were collected, processed, and analyzed *via* LC-MS/MS. A total of 6,230 proteins containing at least one unique peptide were identified with confidence values 99% of FDR estimation. Among all proteins identified, 312 were differentially expressed proteins (DEPs, fold of change >2 or <0.5, *p* value <0.05), including 138 up and 174 downregulated proteins (Figure 1A).

The DEPs were categorized according to the Kyoto Encyclopedia of Genes and Genomes (KEGG) and Gene Ontology (GO) databases, while the enrichment analysis was performed. The KEGG result shows that in HK-2 cells, CBS affects the metabolism of a series of widely different metabolites: glutathione, cysteine and methionine, amino acids, fatty acids, sugars, folate, and purine/pyrimidine (Figure 1B). The GO result shows that among all cellular components, actin cytoskeleton and vesicle are the most affected (Figure 1C). As the protein–protein interaction network and volcano plot have showed, the expression of the actin cytoskeleton was greatly increased upon CBS treatment (Figures 1D,E). Rho GTPases are central regulators of actin reorganization and its subsequent functions (Heasman and Ridley, 2008). The biological process of Rho protein signal transduction was significantly affected by CBS, suggesting an upstream mechanism behind the changes of the actin filament (Figure 1C, Supplementary Figure S1). In contrast to actin, the expression level of the proteins in vesicles was greatly reduced, especially those from the secretory granule (Figures 1D,E). The key proteins of glycolysis were all downregulated by CBS, which includes GAPDH, ENO1, ENO2, PGK1, PGAM1, and TPI1 (Figure 1D). The upregulation of actin and the downregulation of the vesicles and glycolysis were also validated by the enrichment analysis separately using upregulated and downregulated genes (Supplementary Figures S2A,B). The total lists of DEP enrichment analysis results of CBS-treaded HK-2 can be found in Supplementary Data S1.

We then performed GSEA analysis on proteomic data among control and CBS-treated HK-2 cells, in perspective of KEGG (Supplementary Figure S3A). It also shows that CBS downregulated the metabolism of multiple metabolites, including amino acids, fatty acids, folic acid, pyruvate, and glycolysis (Supplementary Figure S3B). In HK-2 cells pretreated with L-buthionine-sulfoximine (L-BSO), GSH was pre-depleted, and such alterations to the metabolism did not occur, suggesting that it is a consequence of the activation of massive GSH biosynthesis (Supplementary Figure S3A). CBS enhanced Notch signaling in HK-2 cells with or without GSH depletion (Supplementary Figures S3A,B). The Notch heterodimer is exposed on the cell surface, which has cysteine-rich Lin Notch repeats (LNR) that could be a potential target of a CBS attack (Weng et al., 2004). Suppression of Notch signaling was found to induce actin accumulation, suggesting another possible upstream mechanism of the increase of actin upon CBS treatment (Wesley et al., 2011). Among the metabolic pathways identified, glutathione metabolism, cysteine, and methionine metabolism and glycolysis have the maximum negative enrichment score (ES), and porphyrin and chlorophyll metabolism has the maximum positive ES (Supplementary Figure S3C). We also performed GSEA analysis in perspective of GOCC, downregulations were found in the ribosome, vesicular lumen, and ER, and upregulation was mainly found in filamentous actin and vesicle tethering complex (Supplementary Figure S3D). The ES of the aforementioned GOCC terms in GSH-depleted HK-2 cells was not as outstanding (Supplementary Figure S3E). The GSEA results of the ribosome, vesicle tethering complex, and actin filament are shown (Supplementary Figure S3F). GSEA results further substantiate the findings in the DEP enrichment analysis, and all GSEA leading-edge proteins in the pathways referred can be found in Supplementary Data S2.

**FIGURE 2:**
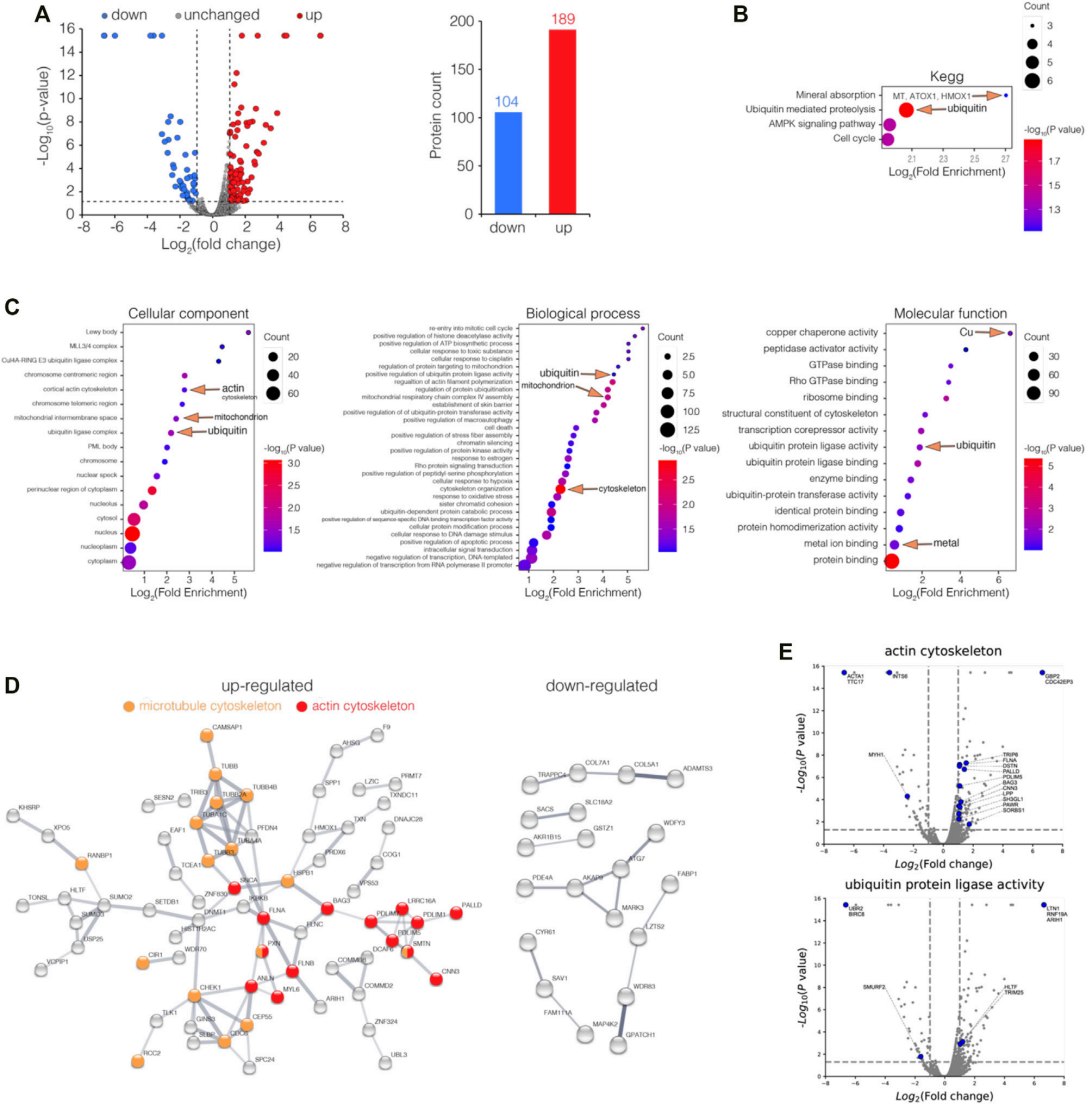
Enrichment analysis of differentially expressed proteins (DEPs) in GSH-depleted HK-2 cells upon bismuth treatment. **(A)** Volcano plot visualizations of DEPs (fold of change >2 or <0.5, *p* value <0.05) in GSH-depleted HK-2 cells treated with 500 μM CBS for 24 h, and 189 and 104 proteins were up and downregulated among 6,230 proteins, respectively. **(B)** Enrichment analysis of DEPs in GSH-depleted HK-2 cells treated with CBS, in perspective of KEGG, and **(C)** GO cellular component, molecular function, and biological process. **(D)** Protein–protein interaction networks of both up and downregulated DEPs generated using STRING. **(E)** Volcano plots visualizing DEPs involved in GO terms: actin cytoskeleton and ubiquitin protein ligase activity. The cells were treated with 500 μM L-BSO for 24 h to remove GSH before CBS treatment. CBS induces MT expression only in the absence of GSH.

To further investigate the bismuth effect on the proteome of HK-2 in the absence of GSH, HK-2 cells were incubated with 500 μM L-BSO for 24 h before being treated with 500 μM CBS for 24 h. The cells were collected, processed, and analyzed *via* LC-MS/MS. Among all proteins identified, 393 were differentially expressed (fold of change >2 or <0.5, *p* value <0.05), including 189 up and 104 downregulated proteins (Figure 2A).

To gain insight into the biological functions affected by bismuth in GSH-depleted HK-2 cells, the DEPs were categorized according to Gene Ontology (GO) and the Kyoto Encyclopedia of Genes and Genomes (KEGG) databases and enrichment analysis was performed. Unlike in HK-2 cells without GSH depletion, bismuth enhanced ubiquitin-mediated proteolysis, suggesting that without GSH, proteins are vulnerable upon bismuth treatment (Figure 2B, Supplementary Figure S2C). Meanwhile, without GSH sequestration, bismuth also greatly affected the chromosome and mitochondrion. The KEGG enrichment analysis shows that bismuth selectively enhanced mineral absorption in GSH-depleted HK-2 cells (Supplementary Figure S2C). Examples of proteins of mineral absorption include MT1E, MT1F, MT2A, ATOX1, and HMOX1, which proves that GSH plays an important protective role in bismuth-treated HK-2 cells before MT.

Furthermore, the DEPs were involved in biological processes including cytoskeleton organization (Figure 2C). The protein–protein interaction network showed a strong interaction network between the set of upregulated proteins which are mainly actin cytoskeleton and microtubule cytoskeleton, and the downregulated DEPs are less well-connected to each other and did not form clique in the graph (Figure 2D). The proteins belonging to GO terms actin cytoskeleton and the ubiquitin protein ligase activity are showed as volcano plots (Figure 2E). The total lists of the DEP enrichment analysis results of CBS-treated HK-2 can be found in Supplementary Data S3.

We also perform GSEA analysis on proteomic data among GSH-depleted HK-2 cells and CBS-treated GSH-depleted HK-2 cells (Supplementary Figure S4). Without GSH sequestration, in perspective of KEGG pathways, bismuth causes severe protein and DNA damage, increased ubiquitin-mediated proteolysis, DNA mismatch repair, and a cytosolic DNA-sensing pathway (Supplementary Figures S4A–C). In perspective of GOCC, bismuth increased the presence of the inclusion body but severely suppressed the levels of proteins from ER and *Golgi* lumen (Supplementary Figures S4D–F). GSEA results further substantiate the findings in the DEP enrichment analysis (Supplementary Figure S2), and all GSEA leading-edge proteins in the pathways referred can be found in Supplementary Data S4.

**FIGURE 3:**
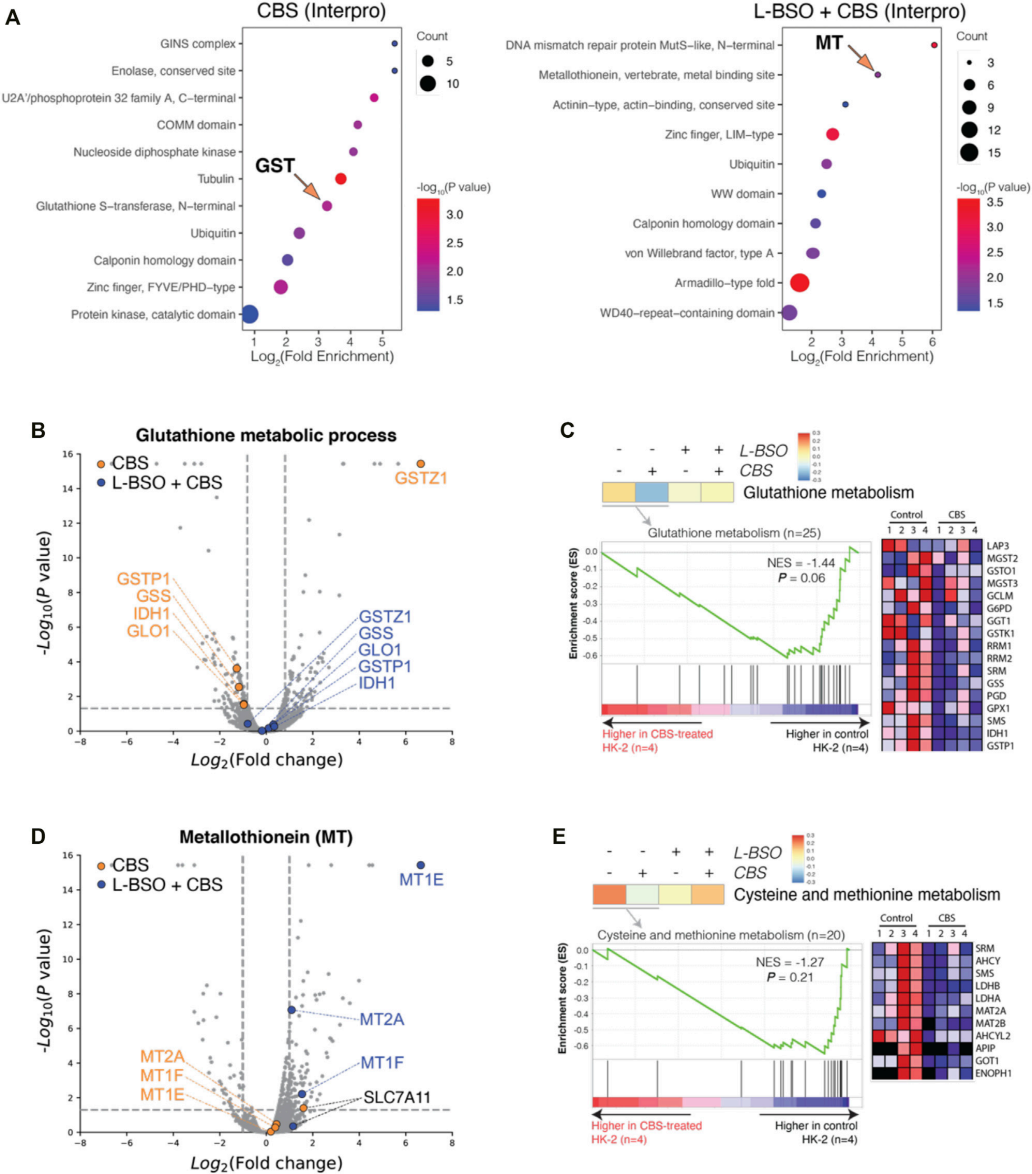
Bismuth induces MT expression only in the absence of GSH. **(A)** Enrichment analysis of DEPs in normal and GSH-depleted HK-2 cells treated with CBS, in perspective of Interpro. **(B)** Volcano plots visualizing DEPs of the GO term glutathione metabolic process. **(C)** GSEA results visualizing the GO term glutathione metabolism. **(D)** Volcano plots visualizing metallothionein isoforms and cystine importer Slc7a11. **(E)** GSEA results visualizing the GO term cysteine and methionine metabolism. HK-2 cells were treated with 500 μM CBS for 48 h or were treated with 500 μM L-BSO for 24 h to remove GSH and then cultured with 500 μM CBS for additional 24 h. Bismuth reduces cisplatin-induced apoptosis and enhances GSH metabolism synchronously.

By comparing the proteomes of control and CBS-treated HK-2 cells, we found alterations mainly toward the major metabolic pathways, including the metabolism of GSH. In GSH-depleted HK-2 cells, the cells underwent severe cytotoxicity but not alteration to major metabolisms. For further investigation, we performed enrichment analysis for HK-2 cells without and with GSH depletion, in the perspective of protein domain (Interpro knowledgebase was used) (Figure 3A). Indeed, Interpro term Glutathione S-transferase, N-terminal, and Metallothionein, vertebrate, and metal binding sites were found outstanding in CBS-treated HK-2 cells without and with GSH-depletion. Proteins GSTZ1, GSTP1, GSS, IDH1, and GLO1 are differentially expressed in normal HK-2 cells but not in HK-2 cells with the L-BSO inhibition of GSH biosynthesis (Figure 3B). GSEA analysis also showed that CBS induced expressional differences in proteins of glutathione metabolism only in HK-2 cells with GSH (Figure 3C). In GSH-depleted HK-2 cells, not HK-2 cells with GSH, the expression levels of MT isoforms MT2A, MT1E, and MT1F were greatly increased (Figure 3D). Cystine is the rare amino acid in the biosynthesis of both GSH and MT; we can see that CBS induced the expression of cystine importer SLC7A11 only when GSH biosynthesis is not suppressed by L-BSO. The metabolism of sulfur-containing amino acids cysteine and methionine was also altered only in normal HK-2 cells (Figure 3E).

**FIGURE 4:**
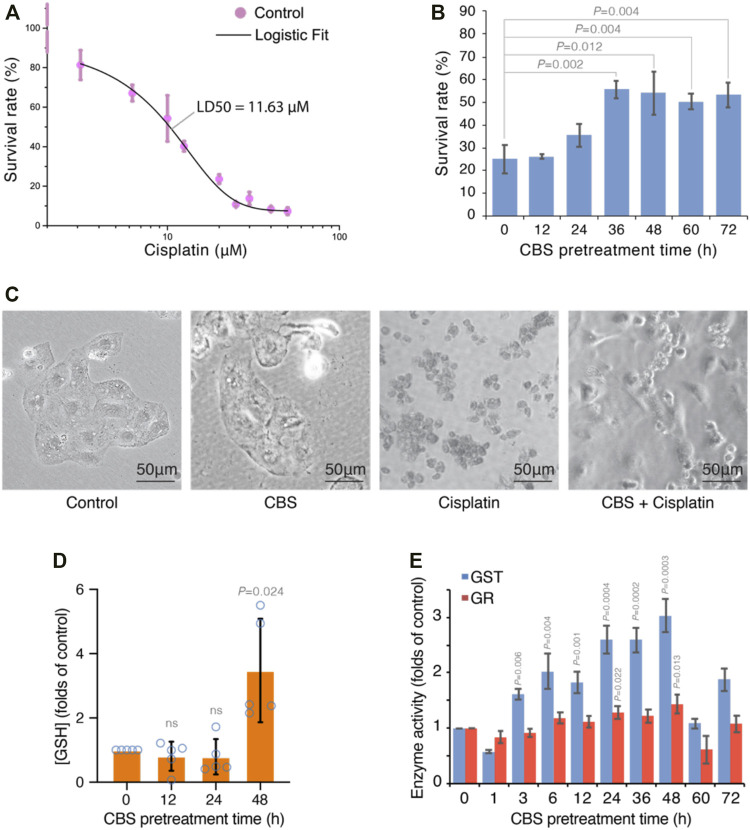
Bismuth synchronously mitigates cisplatin-induced cytotoxicity and enhances glutathione metabolism in HK-2 cells. **(A)** Dose-response curve of cisplatin-induced toxicity in HK-2 cells. The cells were treated with different concentrations of cisplatin for 24 h, and the LD50 is determined to be 11.63 µM by Logistic fit. **(B)** CBS pretreatment mitigates cisplatin-induced cytotoxicity in HK-2 cells. The cells were pretreated with 500 μM CBS for different periods of time before being treated with 20 μM cisplatin for 24 h. **(C)** Bright-field imaging of HK-2 cells under control, cisplatin treatment (20 μM for 24 h), and CBS pretreatment (500 μM for 48 h) plus cisplatin treatment (20 μM for 24 h). **(D)** Bismuth increased the GSH level in HK-2 cells. The cells were treated with 500 μM CBS for 12, 24, and 48 h. **(E)** Bismuth increases GST and GR activities in HK-2 cells. The cells were treated with 500 μM CBS for different periods of time. Bismuth enhances GSH-mediated vesicular sequestration of cisplatin.

In order to study the protective effect of bismuth on cisplatin, we first studied the dose-dependent effect of cisplatin on the human proximal tubular cell line HK-2. A dose–response curve of cisplatin in HK-2 cells was established, and the LD50 is 11.63 μM (Figure 4A). We used 20 μM cisplatin (LD80) in subsequent experiments to evaluate bismuth-induced cytotoxicity mitigation (Figure 4B). HK-2 cells were pretreated with 500 μM colloidal bismuth subcitrate (CBS) for different periods of time. HK-2 cells pretreated with CBS for 36 h or 48 h showed the best protection against cisplatin cytotoxicity, and the cell survival rate increased from 20% to about 50%. The morphology of HK-2 cells without or with CBS pretreatment before cisplatin insult was also imaged, and compared to CBS-pretreated HK-2, the cisplatin-treated cells showed more severe apoptosis (Figure 4C). Glutathione is critical for bismuth uptake and detoxification (Hong et al., 2015). We found that GSH was significantly increased by 2.5 times in HK-2 cells after 48 h of CBS treatment (Figure 4D). The glutathione-S-transferase (GST) and glutathione reductase (GR) activity of HK-2 cells treated with CBS with different periods of time was also increased (Figure 4E), which is consistent with the change in the GSH level (Hong et al., 2015). These data suggest a strong correlation between GSH and bismuth-induced protection against cisplatin toxicity.

**FIGURE 5:**
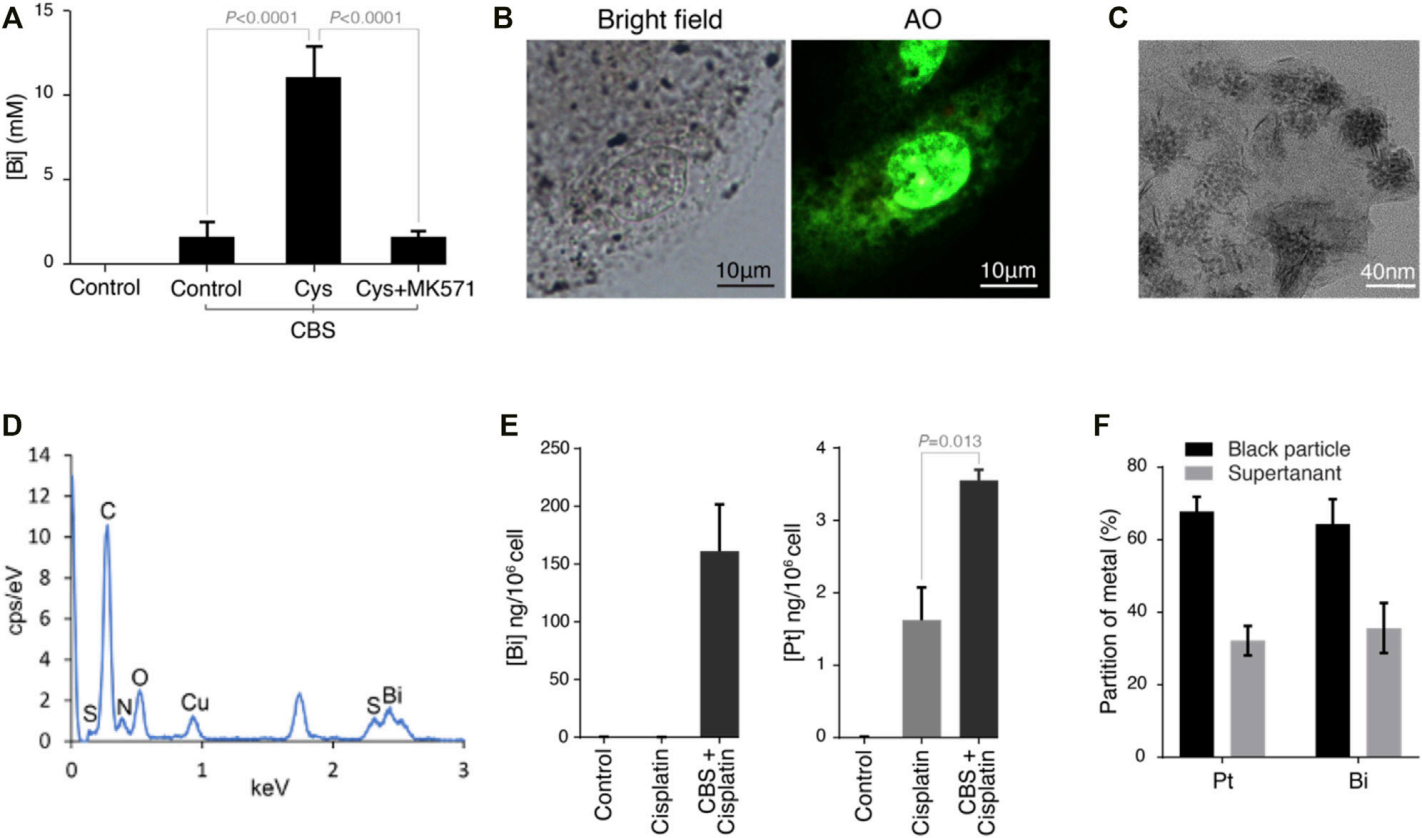
Bismuth enhances the GSH-mediated vesicular sequestration of cisplatin in HK-2 cells. **(A)** Bismuth levels in HK-2 cells treated with control, CBS, CBS with cystine supplement (∼0.1 mg/ml), and CBS with cystine supplement and MK571. **(B)** Bright-field and fluorescent imaging of HK-2 cells treated with 500 μM CBS for 48 h with cystine supplement. HK-2 underwent readily observable darkening. Acridine Orange (AO, 0.5 μM for 30 min) was used to provide background illumination; numerous black particles were thus visualized under a fluorescence microscope. **(C)** Transmission electron microscopy (TEM) images of black particles isolated from CBS-treated HK-2 cells. **(D)** Element identification of black particles using energy-dispersive X-ray spectrometry (EDS), and the element composition is shown in Table 1 **(E)** CBS pretreatment of HK-2 cells (500 μM for 48 h) increased cisplatin absorption. **(F)** More than 60% of both platinum and bismuth are incorporated into black particles in CBS-treated HK-2 cells. HK-2 cells were pretreated with 500 μM CBS for 48 h prior to 20 μM cisplatin cells for 24 h.

In a previous study, we found that bismuth was conjugated to glutathione and then transported into vesicles by an MRP transporter. Since CBS induces the expression of cystine importer SLC7A11 in HK-2 cells (Figure 3D), we further supplied the cells with 0.1 mg/ml of cystine in the culture medium, which dramatically increased the absorption of bismuth (Figure 5A). Furthermore, by inhibition of the MRP transporters (which transport metal-GSH conjugates) using MK571, the absorption of bismuth was greatly reduced. Black particles are synthesized within the vesicles of HK-2 cells after being incubated with CBS. These particles are visualized by the staining of the cell nucleus with Acridine Orange (AO) and imaging with fluorescent microscopy (Figure 5B). We isolate these particles by dissolving away the soluble part of the cell with 1% SDS. The black particles were collected by 15,000 rpm centrifugation for 15 min. The morphology of the particle was determined by transmission electron microscopy (TEM) (Figure 5C). The mean diameter of the particles is approximately 20 nm. We then studied element composition of particles using energy-dispersive X-ray spectrometry (EDS) (Figure 5D). According to EDS result, the atom ratio between bismuth and sulfur is close to 1:1.7 (Table 1). To further investigate whether bismuth pretreatment influences cisplatin absorption and sequestration, HK-2 cells were treated with 500 μM bismuth for 48 h prior to 20 μM cisplatin cells for 24 h. We then quantified the amount of bismuth and platinum in the whole cell, the SDS-dissolved part of the cell, and the particles. The total amount of bismuth absorbed is 150 ng/10^6^ cells (Figure 5E). The platinum content in the cells pretreated with bismuth is around two folds higher than that of the cells treated only with cisplatin. We also compared the amount of bismuth and platinum in the particles and the SDS-dissolved part of cells (supernatant) (Figure 5F). The amount of bismuth and platinum in the particles are both around two times higher than that in the supernatant. These data indicated that both bismuth and platinum are transported to the vesicles and incorporated into the black particles.

**TABLE 1:** Composition of elements in bismuth–sulfur nanoparticles identified using energy-dispersive X-ray spectrometry (EDS).

El	AN	Series	Net unn.C [wt%]	Norm.C [wt%]	Atom.C [at%]	(1 Sigma) [wt%]
C	6	25516	70.48	70.48	86.89	2.18
N	7	2189	3.41	3.41	3.61	0.15
0	8	6066	7.24	7.24	6.70	0.26
S	16	3769	3.73	3.73	1.72	0.15
Cu	29	56144	0.00	0.00	0.00	0.00
Bi	83	4588	15.13	15.13	1.07	1.57
Total			100.0	100.0	100.0	

### Bismuth Reduces Heavy Metal Toxicity by Enhancing Sequestration but not Antioxidation

**FIGURE 6:**
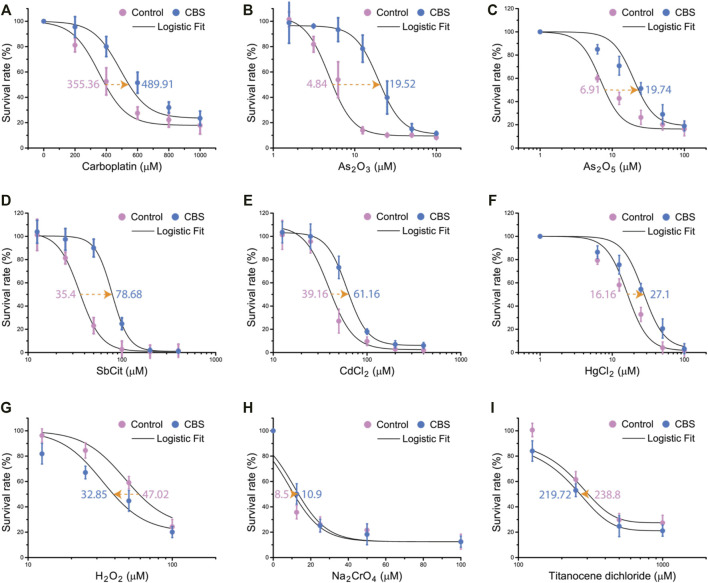
Bismuth mitigates the toxicity of soft metal compounds but not oxidants or hard metal compounds. CBS pretreatment increases the LD50 of HK-2 cells against soft metallodrugs and pollutants: **(A)** carboplatin, **(B)** arsenic trioxide (As_2_O_3_), **(C)** arsenic pentoxide (As_2_O_5_), **(D)** antimony citrate (SbCit), **(E)** cadmium chloride (CdCl_2_), and **(F)** mercury chloride (HgCl_2_). CBS pretreatment did not increase the LD50 of HK-2 against oxidants: **(G)** hydrogen peroxide (H_2_O_2_) and **(H)** sodium chromate (Na_2_CrO_4_). **(I)** CBS did not increase the LD50 of HK-2 against titanocene dichloride. HK-2 cells were treated without or with 500 μM CBS for 48 h, before further treatment with different compounds at different doses for 24 h. LD50s are determined by logistic fit.

The timing of the upregulation of intracellular GSH after CBS treatment concurs that of the reduction of cisplatin toxicity in HK-2 cells and CBS-enhanced cisplatin sequestration to the vesicles, suggesting that GSH plays the major role (compared to MT) against cisplatin toxicity. However, whether the intracellular scavenging of reactive oxygen species or cisplatin by GSH is the main mechanism behind the protective effect of CBS remains unknown. For further investigation, we systematically examined the protective effect induced by CBS on HK-2 cells against a series of soft metal compounds (including anticancer drugs such as carboplatin and arsenic trioxide and common heavy metal pollutants such as cadmium and mercury), hard metal compounds, and oxidant. HK-2 cells were treated with 500 μM CBS for 48 h before being exposed to different compounds. We found that CBS pretreatment only reduced the toxicity of soft metal compounds: carboplatin, arsenic trioxide (As_2_O_3_), arsenic pentoxide (As_2_O_5_), antimony citrate (SbCit), cadmium chloride (CdCl_2_), and mercury chloride (HgCl_2_) (Figures 6A–F). CBS shows no protection against either oxidant hydrogen peroxide (H_2_O_2_) and sodium chromate (Na_2_CrO_4_) or hard metal compounds: Na_2_CrO_4_ and titanocene dichloride (Figures 6G–I). It shows that the CBS-induced protection is general against different heavy metal compounds but not the hard metal compounds and oxidants. It suggests that CBS-induced GSH upregulation mitigates cisplatin toxicity through direct conjugation, instead of reducing cisplatin-induced oxidative stress.

## Discussion

**FIGURE 7:**
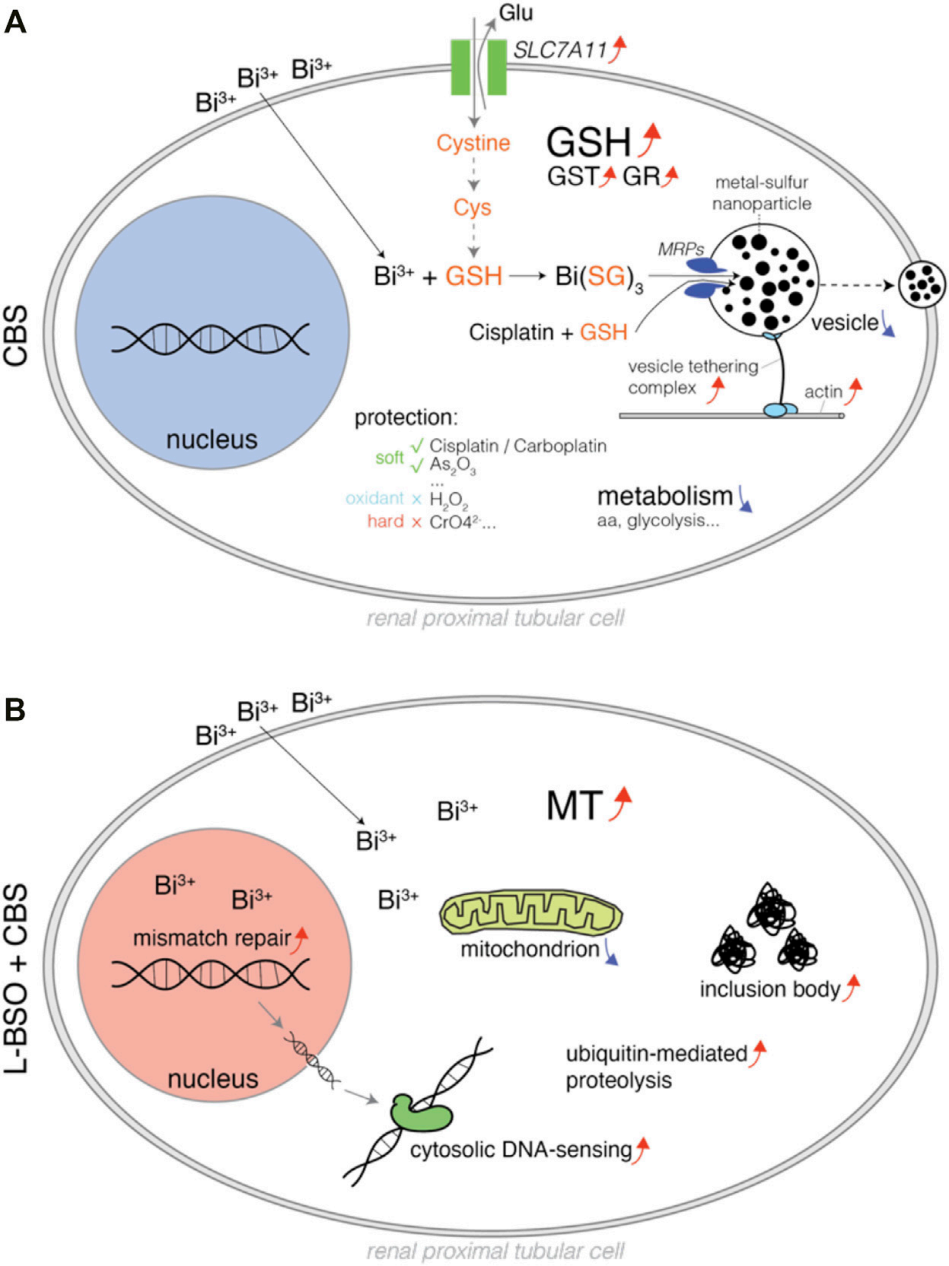
Schematic diagram illustrating the pharmacological, metabolic, and proteomic alterations in bismuth-treated proximal tubular cells. **(A)** HK-2 cells treated with CBS, and the bismuth ions were conjugated to GSH and transported to the vesicles, which can be exported by actin and the vesicle-tethering complex. The mechanism also sequesters cisplatin and likely other soft metal–based compounds. CBS downregulated the metabolism of amino acids and glycolysis but upregulated cystine importer Slc7a11. **(B)** GSH-depleted HK-2 cells treated with CBS, and the MT level was increased. CBS damaged intracellular protein and DNA, causing an increase of inclusion body, ubiquitin-mediated proteolysis, mismatch repair, and cytosolic DNA sensing mechanism. CBS also affects the mitochondrial respirasome.

Cisplatin is one the most important anticancer drugs, which unfortunately causes severe nephrotoxicity. Bismuth drugs were found to mitigate cisplatin nephrotoxicity in rodents. It has been unclear whether GSH or MT plays the major role in bismuth-induced mitigation of cisplatin toxicity. Our evidences suggest that it is GSH that plays the major role, and it is not through oxidative stress reduction but by direct cisplatin sequestration. We systematically profiled bismuth-induced metabolic and proteomic alterations in proximal tubular cells with or without GSH metabolism (Figure 7).

Oxidative stress is one of the most important mechanisms involved in cisplatin toxicity (Dasari and Tchounwou, 2014). Cisplatin was found to induce oxidative stress primarily by targeting the mitochondrion, which affects the protein sulfhydryl group, mitochondrial calcium uptake, and mitochondrial membrane potential (Saad et al., 2004). Cisplatin-induced excessive reactive oxygen species (ROS) damages proteins, lipids, and DNA, causing cell death. Thiol scavenging systems such as superoxide dismutase (SOD), MT, and GSH are critical for the reduction of such damage. SOD did not increase in rats that received Bi^3+^ before cisplatin but in rats that received only cisplatin (Boogaard et al., 1991; Nishikawa et al., 2001; Banci et al., 2012). MT expressions were found elevated in bismuth-treated mouse kidneys (Naganuma et al., 1987; Satoh et al., 1989; Kondo et al., 2004). However, since Pt-bound MT in the kidneys of bismuth-pretreated rats was lower than that of the untreated group, MT was proposed to mitigate cisplatin nephrotoxicity by its antioxidant property (Boogaard et al., 1991). GSH is the most abundant thiol scavenger in mammalian cells, which also antagonizes oxidative stress (Pljesa-Ercegovac et al., 2008; Jos et al., 2009). By testing bismuth-induced protection against different compounds, we find selective protection against soft metal compounds only and not against oxidant or hard metal compounds (Figure 6).

In this report, we found that the induction of MT isoforms (MT1E, MT1F, and MT2A) was found only in HK-2 cells with GSH pre-depletion (Figure 3D). In normal HK-2 cells, no significant increase expression of any MT isoform can be found. Cysteine is the rare amino acid that is required for biosynthesis of MT and GSH, and it is generally generated from the reduction of cystine. Both GSH and MT biosyntheses require cystine; however, bismuth only induces cystine importer Slc7a11 in HK-2 without GSH biosynthesis inhibition (Figure 3D). It suggests that majority of cystine enters the GSH metabolism.

In the animal studies that discovered bismuth’s mitigative effect against cisplatin nephrotoxicity, the timing of bismuth pretreatment was gradually optimized and settled at 48 h prior to cisplatin administration (Kondo et al., 2004; Naganuma et al., 1987; Satoh et al., 1989). We found that bismuth pretreatment takes about 48 h to reach maximal protection against cisplatin-induced toxicity in HK-2 cells (Figure 4). In addition, by feeding quantitative data of bismuth absorption by HK-2 to the SAPT model, we deduce that GSH biosynthesis will be activated after bismuth saturation at 36 h, after which the GSH levels were greatly elevated (Hong et al., 2015). The timing of the onset of bismuth-induced protection *in vivo* and *in vitro* and the timing of the upregulation of GSH levels found *in vitro* and *in silico* agree with each other (Figure 4D) (Morikawa et al., 1990).

Upregulation of GSH also accompanies the upregulation of GST activity, and glutathione S-transferase subunit 3A was also found upregulated in bismuth-treated rat kidney cells (Figure 4E) (Leussink et al., 2003). In addition to ROS scavenging capacity, GSH also binds and transports heavy metal ions to vesicles or extracellular spaces (Hong et al., 2015; Rosen, 1999; Mielniczki-Pereira et al., 2008). GST catalyzes the conjugation of GSH to xenobiotics, and its upregulation suggests that GSH conjugation to cisplatin can be greatly enhanced. This is further proved by TEM-EDS and ICP-MS experiments (Figure 5). The transportation of GSH-metal conjugates is mediated by MRP/CFTR family transporters such as Ycf1p and MRP1. With abundant cystine supplement, the sequestration of bismuth by glutathione in HK-2 cells forms a large number of black nanoparticles within the vesicles (Hong et al., 2015). Formation of such particles was inhibited by the MRP1 inhibitor MK571 (Figure 5A).

Because of the robust intracellular GSH scavenging system, human cells can endure high concentrations of bismuth (Hong et al., 2015). It results in drug selectivity against pathogens that cannot synthesize GSH, such as *Helicobacter pylori*, making bismuth a member of clinical quadruple therapy against *H. pylori*. In proteomic profiling, we found that bismuth increases the vesicle-transporting system, enhanced expression of actin, vesicle tethering complex, and vesicle exocytosis (Figure 1, Supplementary Figure S1). Bismuth suppresses all major metabolic pathways, including the metabolism of amino acids, fatty acids, purine and pyrimidine, folate, and energy. Overall, tune-down might reduce the untoward effects of cisplatin on all these pathways. Specifically, bismuth reduces the amount of ribosome and protein-folding complexes, suggesting a decreased protein biosynthesis. Bismuth reduced energy metabolic pathways such as glycolysis, pyruvate metabolism, and TCA cycle. Heme oxygenase-1 (HMOX1) safeguards the cellular redox balance and has been demonstrated to reduce cisplatin-induced nephrotoxicity by safeguarding cellular redox, induction of which could also be a part of the bismuth-induced mitigative effect (Agarwal et al., 1995; Schaaf et al., 2002). It was found that single high doses of bismuth can trigger the redistribution of N-cadherin and the disruption of homotypic cell adhesion (Leussink et al., 2001). In this report, we found that bismuth increases cell–cell adhesion and integrin activation. Indeed, we did find that HK-2 cells adhere tighter to the culture plate after a period of time of bismuth treatment, and the cells are harder to be detached by trypsin.

Many subtypes of renal cells have been recently identified due to their topologic, metabolic, and transcriptional phenotypic differences, and further details and patterns surely exist behind cisplatin nephrotoxicity (Muto et al., 2021). Renal proximal tubular cells are known to possess vulnerability upon cisplatin insults, but other types of renal cells have not been systematically evaluated. Since ROS signaling regulates the renal function along the nephron, even though it might not be the case for proximal tubular cells, its effects on other renal cell types are still worth further study. Application of single-cell methodologies such as single-cell RNA-seq, single-nucleus RNA-seq, and mass cytometry would reveal much more information in future studies (Wilson et al., 2019).

## Data Availability

The datasets presented in this study can be found in online repositories. The names of the repository/repositories and accession number(s) can be found in the article/Supplementary Material.
