# Supplementary material for: Bismuth Reduces Cisplatin-Induced Nephrotoxicity Via Enhancing Glutathione Conjugation and Vesicular Transport
Source: Front Pharmacol. 2022 Jun 16;13:887876. doi: 10.3389/fphar.2022.887876 (PMC9243339; doi:10.3389/fphar.2022.887876)
Supplement: Supplementary file 1 [file DataSheet1.pdf]

Supplementary Materials for

Bismuth reduces cisplatin-induced nephrotoxicity via enhancing glutathione conjugation and vesicular transport

Hui Jiang[1], Yifan Hong[2], Guorong Fan[3]

[1] Tongji University School of Medicine, Shanghai 200092, China.
[2] Institute of Molecular Physiology, Shenzhen Bay Laboratory, Shenzhen 518132, China.
[3] Tongji University School of Medicine, Shanghai 200092, China; Department of Clinical Pharmacy, Shanghai General Hospital, School of Medicine, Shanghai Jiao Tong University, Shanghai 200025, China.
These authors contributed equally: Hui Jiang, Yifan Hong.
Correspondence: Yifan Hong: angivan@163.com, Guorong Fan: guorfan@163.com

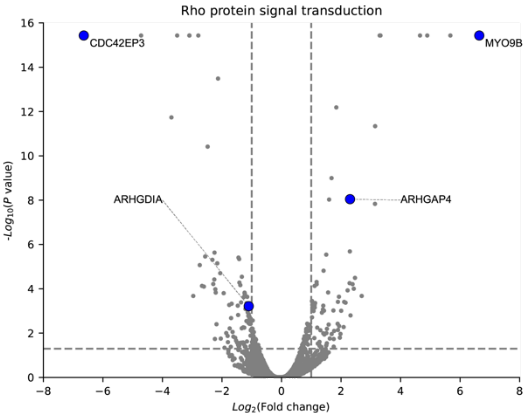

Fig. S1. Volcano plots visualizing DEPs of GO:0007266 Rho protein signal transduction in CBS-treated HK-2 cells.

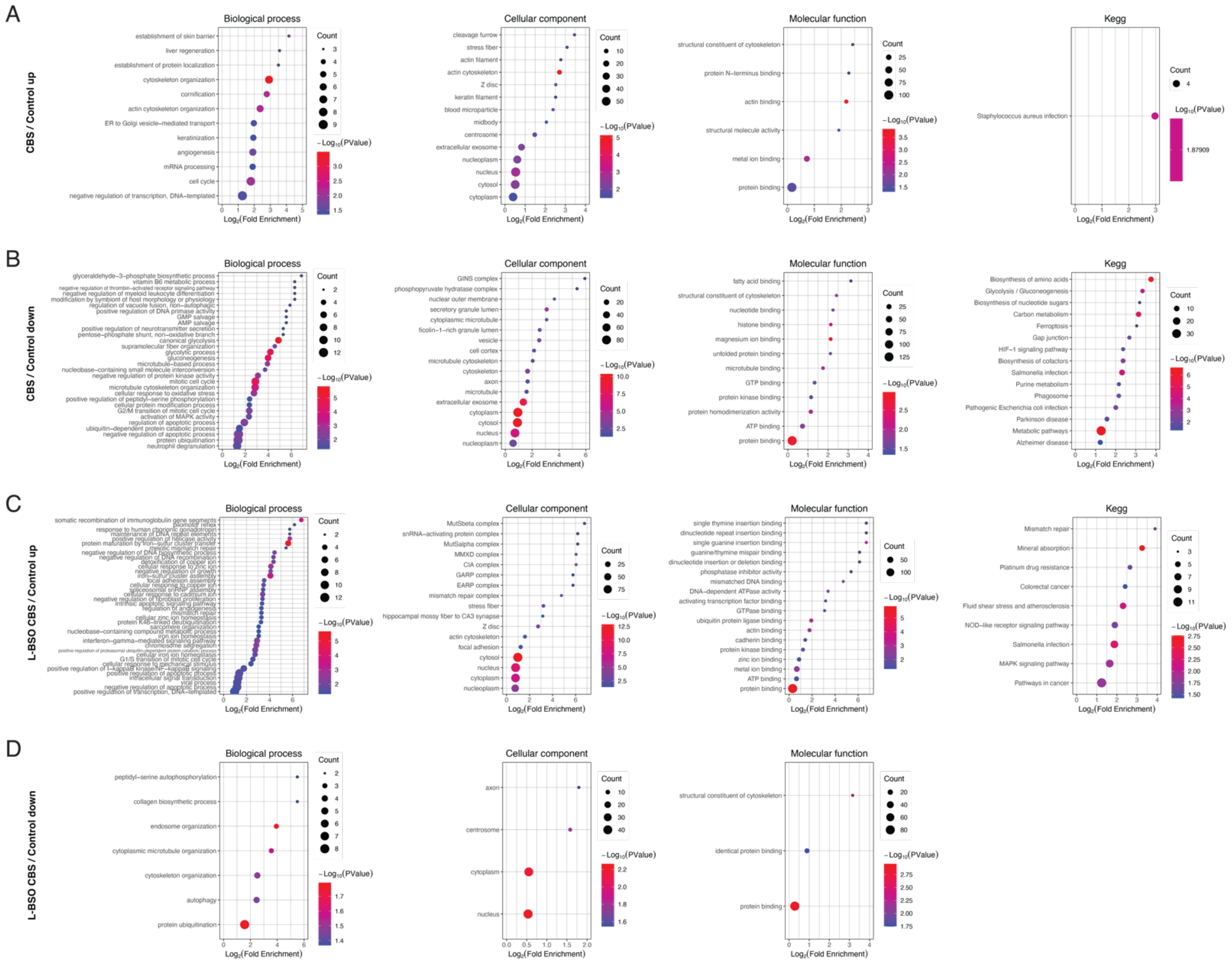

Fig. S2. Enrichment analysis of differentially expressed proteins (DEPs) in CBS-treated HK-2 cells. Enrichment analysis in perspective of Kegg, GO cellular component, molecular function and biological process of (A) up-regulated and (B) down-regulated DEPs in CBS-treated HK-2 cells, (C) up-regulated and (D) down-regulated DEPs in CBS-treated GSH-depleted HK-2 cells. The cells were treated with 500 μM CBS for 48h, or were treated with 500 μM L-BSO for 24h to remove GSH and then cultured with 500 μM CBS for extra 24h.

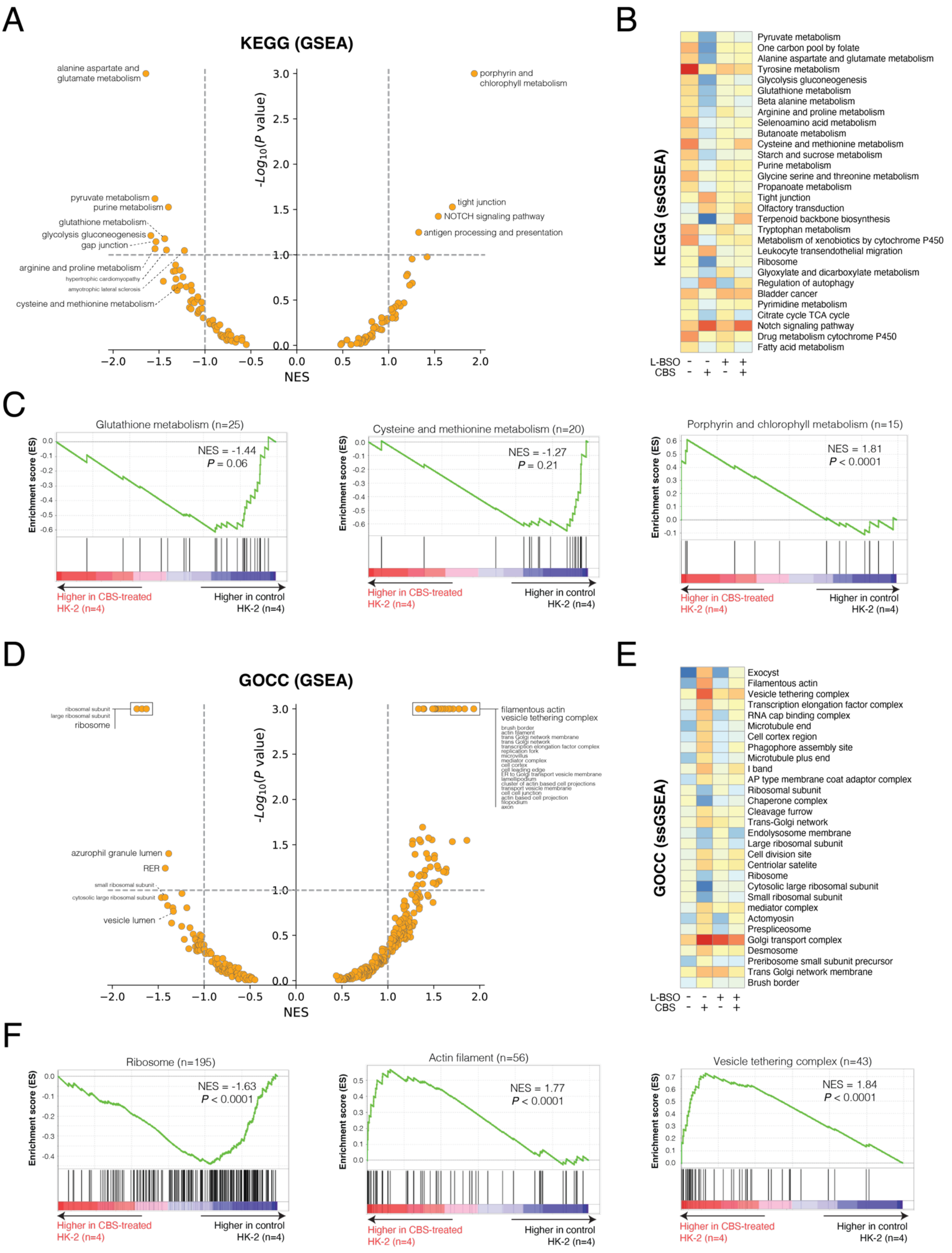

Fig. S3. GSEA analysis of proteomic changes in CBS-treated HK-2 cells.

(A) Kegg GSEA analysis of proteins from control and CBS-treated HK-2 cells. (B) Heatmap of alterated pathways in perspective of Kegg (ssGSEA) from control and CBS-treated HK-2 cells. Color of each cell represents the average ssGSEA enrichment scores of that pathway; red denotes activation and blue denotes suppression. White cells represent non-enrichment. HK-2 cells treated with 500 μM L-BSO for 24h to remove GSH were used for comparison. (C) GSEA results visualizing Kegg pathways: glutathione metabolism,

cysteine and methionine metabolism and porphyrin and chlorophyll metabolism. (D) GOCC GSEA analysis of proteins from control and CBS-treated HK-2 cells. (E) Heatmap of alterated pathways in perspective of GOCC (ssGSEA) from control and CBS-treated HK-2 cells. (F) GSEA results visualizing GOCC terms: ribosome, actin filament and vesicle tethering complex.

The cells were treated with 500 μM CBS for 48h, or were treated with 500 μM L-BSO for 24h to remove GSH and then cultured with 500 μM CBS for extra 24h.

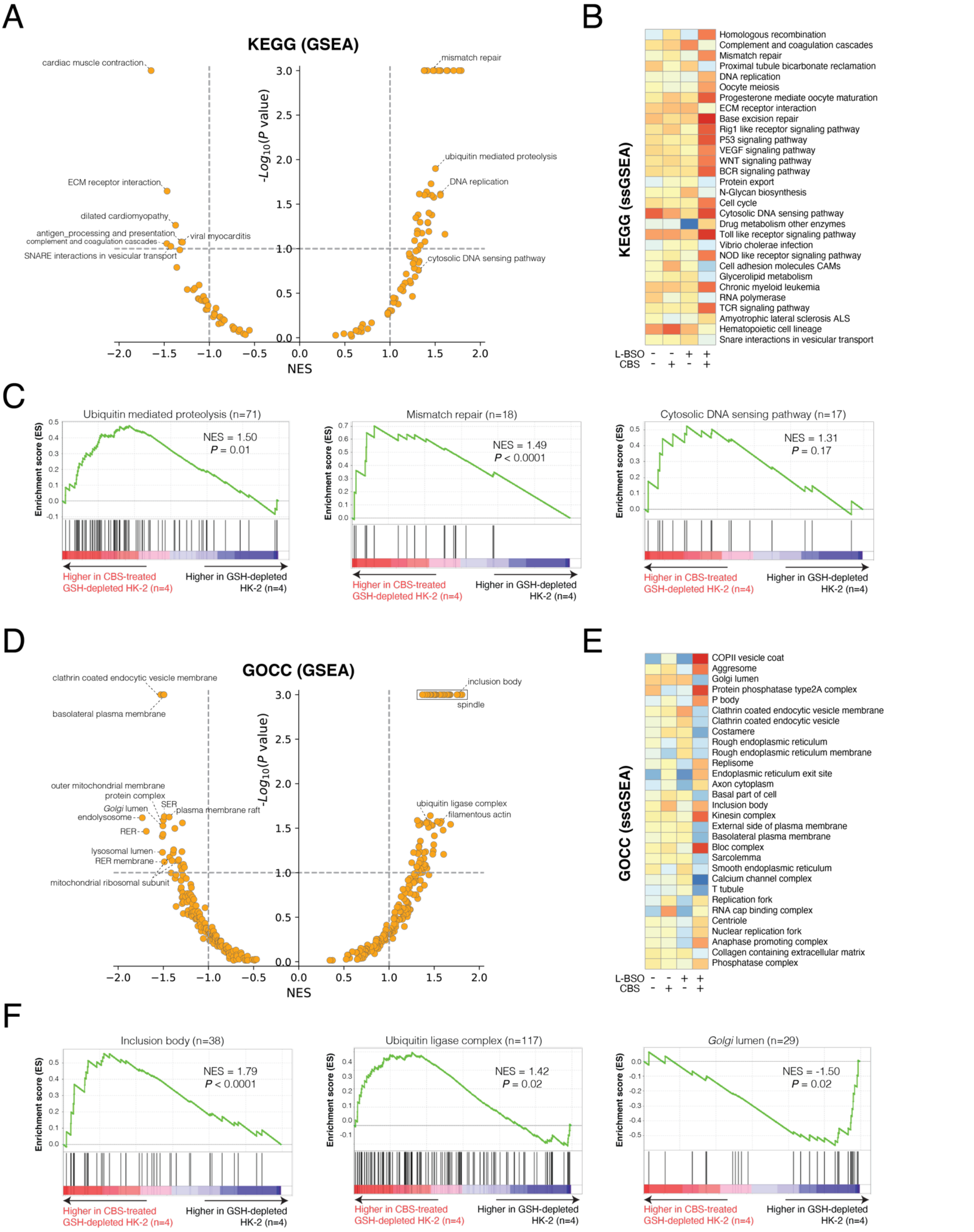

Fig. S4. GSEA analysis of proteomic changes in CBS-treated GSH-depleted HK-2 cells.

(A) Kegg GSEA analysis of proteins from control and CBS-treated GSH-depleted HK-2 cells. (B) Heatmap of alterated pathways in perspective of Kegg (ssGSEA) from control and CBS-treated GSH-depleted HK-2 cells. Color of each cell represents the average ssGSEA enrichment scores of that pathway; red denotes activation and blue denotes suppression. White cells represent non-enrichment. CBS-treated HK-2 cells were also compared. (C) GSEA results visualizing Kegg pathways: ubiquitin mediated proteolysis, mismatch repair and cytosolic DNA sensing pathway. (D) GOCC GSEA analysis of proteins from control and CBS-treated GSH-depleted HK-2 cells. (E) Heatmap of alterated pathways in perspective of GOCC (ssGSEA) control and CBS-treated GSH-depleted HK-2 cells. (F) GSEA results visualizing GOCC terms: inclusion body, ubiquitin ligase complex and *Golgi* lumen.

The cells were treated with 500 μM CBS for 48h, or were treated with 500 μM L-BSO for 24h to remove GSH and then cultured with 500 μM CBS for extra 24h.
